# Towards a Postural Indicator of Back Pain in Horses (*Equus caballus*)

**DOI:** 10.1371/journal.pone.0044604

**Published:** 2012-09-07

**Authors:** Clémence Lesimple, Carole Fureix, Emmanuel De Margerie, Emilie Sénèque, Hervé Menguy, Martine Hausberger

**Affiliations:** 1 Université de Rennes 1, Laboratoire d’éthologie Animale et Humaine EthoS - UMR CNRS 6552, Station biologique, Paimpont, France; 2 Université de Rennes 1, Laboratoire d’éthologie Animale et Humaine - UMR CNRS 6552, Campus de Beaulieu, Rennes, France; 3 Chiropractic Practice, St Jacques de la Lande, France; Université Pierre et Marie Curie, France

## Abstract

Postures have long been used and proved useful to describe animals’ behaviours and emotional states, but remains difficult to assess objectively in field conditions. A recent study performed on horses using geometric morphometrics revealed important postural differences between 2 horse populations differing in management conditions (leisure horses living in social groups used for occasional “relaxed” riding/riding school horses living in individual boxes used in daily riding lessons with more constraining techniques). It was suggested that these postural differences may reflect chronic effects of riding techniques on the horses’ kinematics and muscular development. In the present study, we tried to evaluate the interest of postural measures to assess welfare in horses. This study was separated into 2 parts. First, 18 horses coming from these 2 types of populations (leisure/riding school horses) were submitted to 2 back evaluations by 1) manual examination (experienced practitioner) and 2) sEMG measures along the spine. We then measured neck roundness on 16 of these 18 horses. The results highlighted high correlations between manual and sEMG examinations over the spine. sEMG measures at the different locations were strongly correlated all over the spine. Moreover, neck postures and muscular activities were strongly correlated, horses with concave necks having higher sEMG measures both at precise locations (*i.e.* cervical sites) but also when comparing neck postures to the whole spine muscular activity highlighting the functioning of horses’ back as a whole. Lastly, strong differences appeared between the populations, leisure horses being evaluated as having sounder spines, exhibiting lower sEMG measures and rounder neck than the riding school horses. sEMG measures and neck “roundness” seemed therefore to be reliable indicators of back disorders, easy to evaluate in field conditions. This highlights the accuracy of using postural elements to evaluate the animals’ general state and has important implications for animals’ welfare evaluations.

## Introduction

Postures have long been used and proved useful to describe animals’ behaviours and emotional states (*e.g.* [1], [2]). More recently, several studies have proposed to include them in welfare assessment (*e.g.* [2], [3]). However, posture assessment is still based on few salient elements. Estimation of anxiety level in mice is thus mostly based on the trunk and tail angles [4], while ear and tail postures are used in sheep to evaluate the emotional value of given situations [2]. When used, global posture assessments are often based on very coarse postural elements, e.g. the animal is merely recorded as lying or standing [5], [6], [7], or remain subjective (e.g. the low posture in stressed dogs, where “the position of the tail is lowered (…) and the legs are bent” compared to “the breed specific posture shown by dogs under neutral conditions”, [3]).

One main problem however to make postures reliable tools for such an assessment is the difficulty to develop repeatable, objective and comparable measures. Postures are generally characterized on the basis of a few elements (*e.g.* head and tail) and only evaluated by mere visual inspection. The use of anatomical landmarks has made objective and reproducible measures possible but most such studies require highly standardized and artificial situations [8], [9], [10]. If postures are to be a useful tool for welfare assessment, their measure needs to be possible in the home environment of the animal and should lead to few reliable but clearly visible markers.

A recent study performed on horses and using the technique of geometric morphometrics has revealed that postures could be measured by this approach in the animals’ usual environment [11]. The comparison of two horse populations, one composed of leisure horses living outdoors in stable social groups and used in occasional (weekly) “relaxed” (long reins) riding and the other of riding school animals living in single stalls and submitted to daily “constrained” riding (see [12]), revealed important differences between them, in terms of postures both while standing and being led in hand. The results showed that, amongst more global postural differences, the outdoor population showed rounder backs and necks. It was suggested that these differences may reflect environmental conditions, such as a potential impact of beginner riders’ hands actions on the horses’ neck postures at work [12], leading to chronic effects on kinematics and muscular development (*e.g.* [13], [14]). Horses with back problems often show flat/rigid backs [9] and tend to hold their head high [15]. Factors such as chronic “psychological” stress may induce hollowness, and modifications of postures through tensions. In humans, anxiety is known to tense up muscles [16].

In the present study, we tried to evaluate the interest of postural measures to assess welfare in horses, an interesting animal model that shares with humans potential physical and psychological stress at work (*e.g.* [17], [18]), in addition to restricted life conditions (social and spatial restrictions, diet disturbance… *e.g.* [19], [20]). On the basis of the former exploratory study [11], we concentrated on the degree of “roundness” of the neck region, which was one of the most striking differences between differently managed populations. As vertebral problems have a very high prevalence in horses and can be caused by a wide range of lesions (*e.g.* [12], [21], [22]) it seems quite interesting to be able to detect, through simple postural elements, potential painful chronic problems. The use of radiographic imaging is limited by the thickness of the surrounding soft tissues [23], ultrasonic and scintigraphic approaches have their use but remain difficult to apply in field conditions, on large samples of horses [23], [24]. Some earlier studies therefore had been based on practitioners’ evaluations [12], [22]. However, it is not possible on large samples to have a practioner “at hand” and the need for clear comparative data led us to use surface electromyographic (sEMG) measures.

Growing literature suggests significant differences in muscular activity between LBP and healthy people and sEMG measures seem to convey these differences (see [25] for a review). Several studies highlighted that sEMG measures at rest enables the detection of various muscular dysfunctions or hyperactivity [26] and LBP patients had higher sEMG levels than healthy controls during different posture patterns [27], [28]. Recently, veterinarians specialized in horses’ vertebral health began to use EMG devices to explore horses’ back functioning during movements [29], [30], but to our knowledge, no such assessment was ever performed in order to detect chronic back disorders.

The present study is therefore separated into two parts: 1) validating the use of sEMG as an alternative to manual evaluation of potential vertebral disorders throughout the axial skeleton, 2) relying neck postures to sEMG measures as reflecting back problems (cervical but also all over the spine). Two populations, “extremes” in terms of management and riding techniques, were compared as in Fureix *et al.* [11]: leisure horses living in social groups outdoors and riding school horses living in single stalls. The aim here was to validate further postures as tools for welfare measurement, not to disentangle the factors responsible for potential problems.

## Animals and Methods

Experiments complied with current French laws (Centre National de la Recherche Scientifique) related to animal experimentation and were in accordance with the European directive 86/609/CEE. No licence/permit/institutional ethical approval was needed. Animal husbandry and care were under the management of a private owner (study 1) or the riding school staff (study 2). This experiment involved only horses in the “field” (no laboratory animals). We studied two samples of horses kept under different conditions to investigate the reliability of sEMG measures in reflecting back disorders in study 1 and the relation between sEMG measures and neck postures in study 2.

### Horses

**Figure 1:**
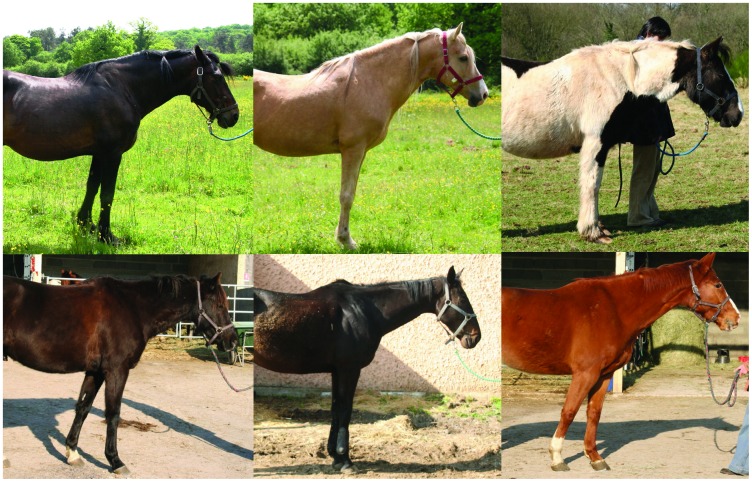
Example of 3 Group 1 horses (on the top of the figure) and 3 Group 2 (at the bottom of the figure) horses when standing.

The evaluations were performed on horses, distributed into two groups (**Fig. 1**).

The first group corresponded to 9 domestic horses (2 mares, 4 stallions and 3 geldings; 10 to 26 years old, ±es = 19.5±2.1) kept under natural conditions in stable social groups for several years, in 1–2 ha natural pastures, fed grass and hay ad libitum during winter (no industrial pellets) and used for occasional leisure outdoor “relaxed” riding (with long reins). They lived in 3 groups in the same site (Group 1).

The second group corresponded to 9 riding school horses (1 mare and 8 geldings; 12 to 21 years old, ±es = 16.4±0.9). These horses were kept in 3×3 m individual straw-bedded boxes, fed industrial pellets three times a day and hay once a day, exercised in riding lessons for 4–12 h per week with more constraining techniques. All horses were in the same riding school and had at least one free day per week(Group 2).

### Back Evaluation

#### Chiropractic examination (see also [12], [22])

The evaluation of the study horses’ spine was performed by a 20 years experienced licensed chiropractor who was totally blind to the results of the electromyogram and did not know the horses beforehand. Examination was based on bony and soft tissue manual palpation for localised regions of vertebral stiffness based on spinal mobilisation and palpable areas of muscle hypertonicity [31], [32] and have been shown to be efficient to detect back pain [33], [34]. Manual palpation was performed from head to tail in each horse’s box outside working hours. The horse was slightly restrained by an unfamiliar experimenter (MH) who was also blind to the other data (did not participate to sEMG recordings). Data included the proportion of vertebrae affected, and horses were classified into 3 categories: totally exempt, slightly affected (1 vertebral site affected) and severely affected (more than one vertebral site affected out of the 7 cervical, 18 thoracic, 6 lumbar, 5 sacral and 15 coccygeal vertebral sites present in horses).

Data reliability was assessed by a second evaluation performed respectively by a veterinarian specialized in osteopathy in Group 1 and a second chiropractor in Group 2 whose techniques of detection (if not in usual care) were similar in the present study. The Kappa agreement (Kappa index, [35]) conducted on vertebral sites affected was respectively of 100% and 97.71% respectively.

All the chiropractic evaluations were performed for free by H. Menguy himself, manager and only employee of the chiropractic practice. Moreover the manual palpations were carried on Sundays, outside working time of the practice.

#### Static surface electromyogram

**Figure 2:**
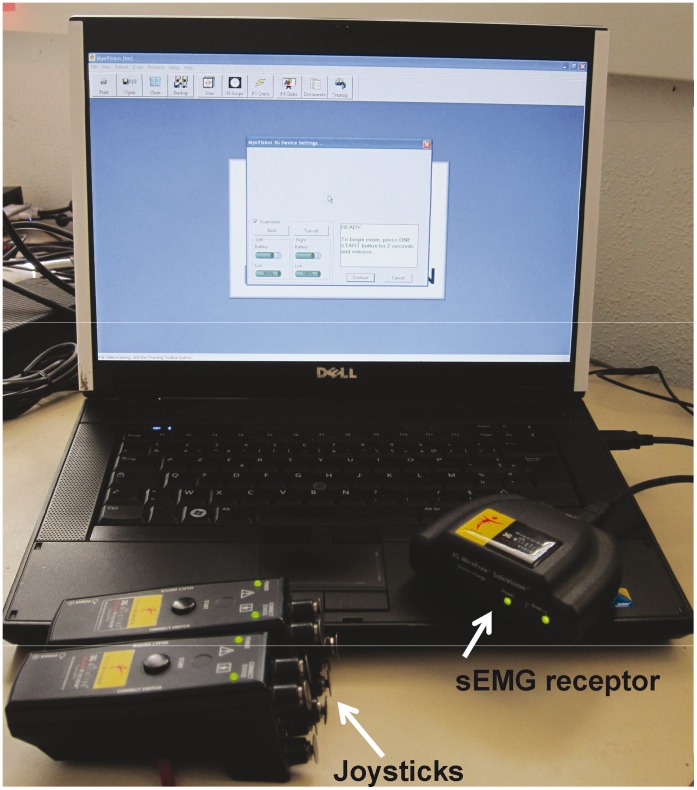
Myovision® sEMG device. The 2 joysticks are on both sides of the spine, and data are recorded via the receptor linked to the computer.

**Figure 3:**
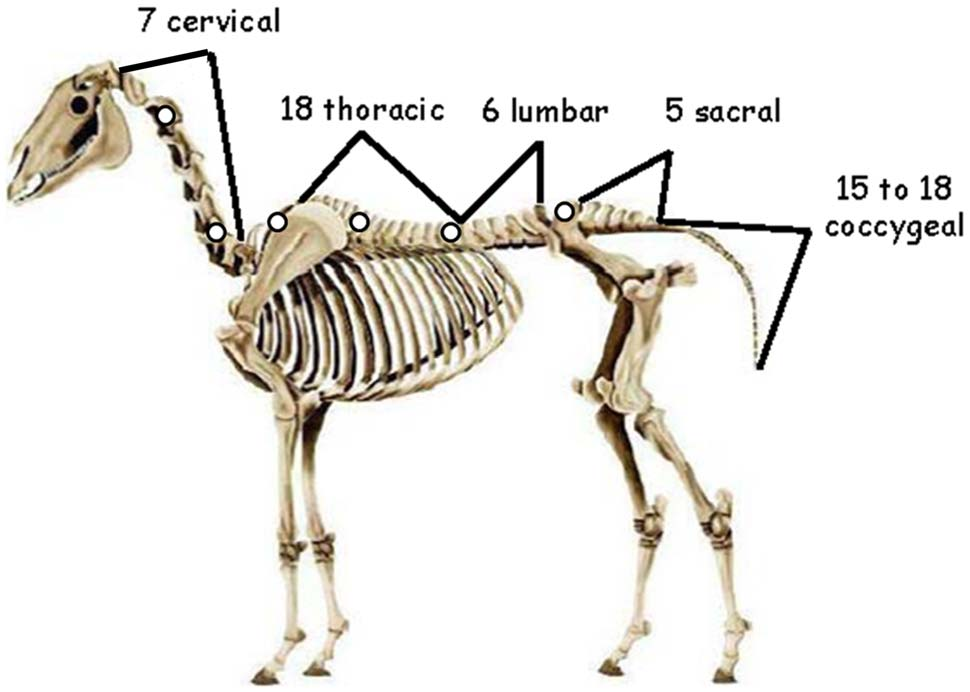
Representation of a horse skeleton with the locations of electrodes for sEMG measurements. The electrodes were placed at the level of the white spots of the figure.

**Figure 4:**
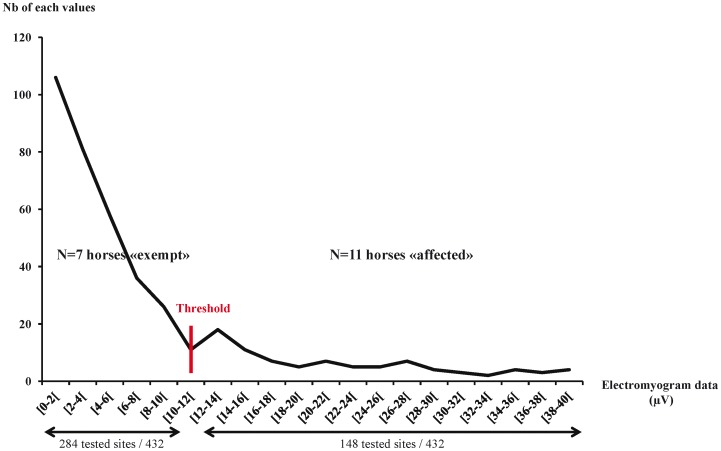
Distribution of sEMG measures across the number of tested sites concerned and threshold value. All sEMG data of the population were pooled (N = 2 groups, 18 horses × 12 tested sites × 2 sides of the spine).

**Figure 5:**
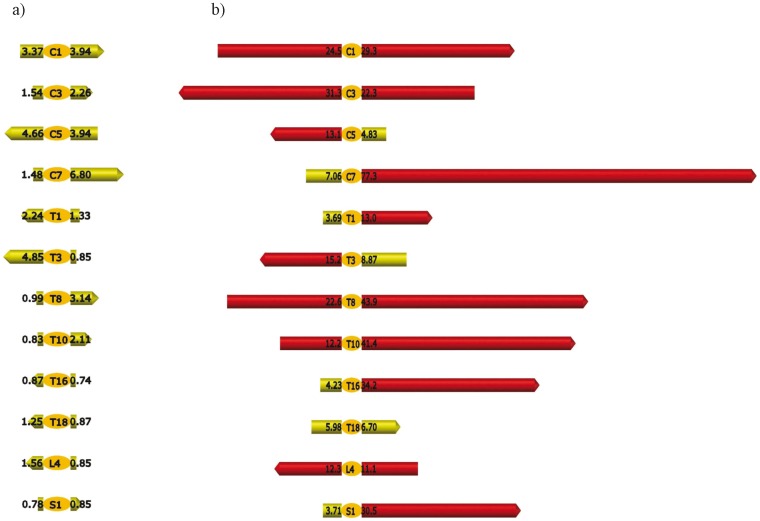
Examples of sEMG signals: representations of values along the spine. a) sEMG signal for a sound horse. The exact values of muscular activity are presented for each tested site in µV (all values <10 µV). Values under 10 µV are represented in green. b) sEMG signal for a severely affected horse with 5 tested sites affected. The exact values of muscular activity are presented for each tested site in µV Values under 10 µV are represented in green and values over 10 µV are represented in red. The five affected sites present a muscular activity over 10 µV on both sides of the spine (C1, C3, T8, T10, L4).

The sEMG examinations were conducted by a second experimenter (CL), blind to the results of the manual palpation (not involved in the chiropractic evaluations), using a wire free device (Myovision®). The experimenter had 2 joysticks with 5 electrodes on each, designed to record muscle activities at the level of the vertebrae before and at the vertebrae after the joystick location. Muscular activities recorded were sent to a receptor related to a computer (**Fig. 2** **)**. The two joysticks were placed at the level of C2, C6, T3, T9, T17, and L6 **(** **Fig. 3** **)** one on each side of the spine, and electrodes gave the muscular activities at the level of C1, C3, C5, C7, T1, T3, T8, T10, T16, T18, L5 and S1. Thus we obtained muscular activity all along the neck, at the level of the shoulder, at the basis of the withers, at the level of the thoracolumbar joint and at the level of the lumbosacral joint, which are reported in the literature as very likely to be affected by musculoskeletal lesions (*e.g.* [34]). The raw sEMG values were used (µV, see [38]). Most muscular activities recorded along the spine (432 tested sites: 18 horses * 12 sites *2 sides of the spine) were low (<10 µV) while a consistent number of residual tested sites showed activities between 10 and 40 µV (**see** **Fig. 4** **.**). The curve of sEMG values and number of sites tested (**Fig. 4** **.)** revealed a threshold at 10 µV which therefore was considered a value from which the tested site (taking both sides into account: Left and Right ≥10 µV) was considered as “hyperactive” (here called “affected”). Manual palpation only allowed categorical classification of horses (0, 1 or more affected vertebrae) as it was impossible for the practitioners to have precise comparative evaluations (no numerical values). Therefore results of sEMG values were replaced in the same categories: totally exempt (**Fig. 5a**), slightly affected (1 site affected) and severely affected (at least 2 sites affected) (**Fig. 5b**).

In the two groups, examinations were performed on a flat ground, outside the pasture in the first group, and in the corridor of the stable in front of each horse’s box in the second group, outside any noise or disturbance (working activity, people around…). The experimenter (the same for all horses, CL) paid attention to the horses’ feet positions: anterior and posterior feet were on a line**.** Horses were kept motionless, slightly restrained with a rope, by a second experimenter.

### Neck Posture Measurements

Seven of the 9 group 1 horses and all group 2 horses were also involved in posture measurements.

Horses were observed while interacting with an experimenter: walking and standing motionless near the experimenter (the same 2.6 m long and 600 g lead rope was used in all interactions). The experimenter did not talk to the horse, stayed on its left side and held the rope slackly at a predefined distance from the horse’s head (1 m), so that the experimenter never pulled the rope or the horse’s head. Horses’ postures were recorded using photographs taken perpendicularly 10±1 m from the horse (digital camera Canon EOS 20D, zoom lens 50 mm to limit perspective distorsions). All data were recorded by the same experimenters (E.S taking pictures, C.F. leading horses). Data recording took place between 08.00 AM and 06.00 PM during a two days period in both groups (during quiet time, with no riding lessons in the riding school). Leisure horses were photographed 10 times when standing motionless near the experimenter, and 20 times when walking. Horses in riding schools were less available (involved in riding lessons) and could be photographed on average 2.4±0.8 times when standing and 4.5±0.9 times walking.

Five markers (self adhesive red felt discs, 34 mm in diameter, visible on all coat colours) were stuck onto the horses’ right side, in accordance to Fureix et al. [11]. The landmarks were placed in a sagittal plane in relation to skeletal or muscular cues (enabling consistent reproduction of positioning) on the neck and head of the horses. Landmarks were placed on: the cervico-thoracic junction (Marker 1, M1); the trapezium cervical ligament at the level of C3 (Marker 2, M2); the dorsal aspect of the wing of the atlas (Marker 3, M3); the temporomandibular joint (Marker 4, M4) and on the rostral aspect of the facial crest (Marker 5, M5). These positions were proved to be efficient to assess horses’ posture when standing and walking, led by an experimenter [11].

**Figure 6:**
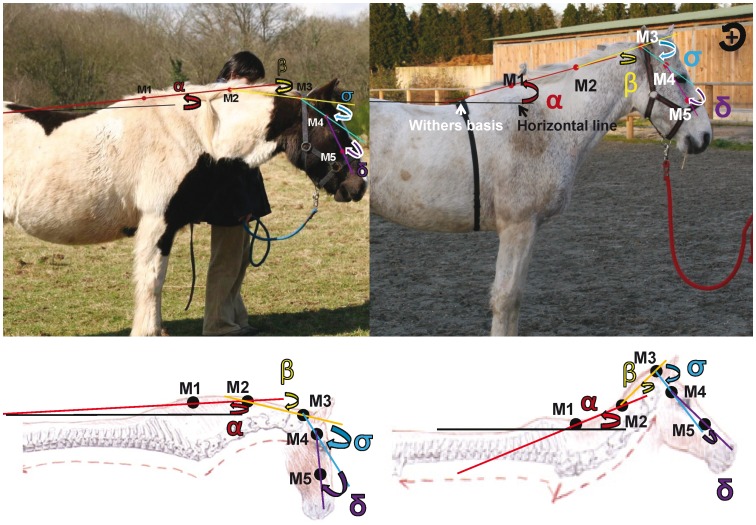
Representation of angles for neck posture measurement (Horse kept under natural conditions on the left and riding school horse on the right). α represents the neck’s elevation, β represents the neck’s curve and γ represent the M3–M5 angle (head-neck angle).

In order to quantitatively evaluate neck height and roundness, different angles were measured, using usual trigonometrical rules (home-made worksheet, EDM) (**Fig. 6**):

Angle α: Formed by the segment (M1–M2) and the horizontal plan running by the withers’ basis (lowest point of the withers, between withers and back). It represents the neck’s elevation [39]: the more elevated the neck was, the more the angle was positive, and the less elevated the neck was, the more the angle was negative.
Angle β: Formed by the segment (M1–M2) and the segment (M2–M3). It represents the neck’s curve: the more concave the neck was, the more the angle was positive, and the rounder the neck was (cervical flexion), the angle was negative.
Angle σ: Formed by the segment (M2–M3) and the segment (M3–M4).
Angle δ: Formed by the segment (M3–M4) and the segment (M4–M5).

The last two angles were pooled (angle γ) to represent the M3–M5 angle (between the atlas and the rostral aspect of the facial crest). The narrower the head-jaw angle was, the more the angle was negative.

The angles measurements required the horses to be exactly perpendicular to the camera. In order to have the same number of photographs per horse, 2 photographs were analysed for each horse while it was standing and 4 while walking (2 when the horse’s neck was in the highest and 2 when it was the in lowest position, called here “high walking point” and “low walking point”). Photographs were taken in series of burst-shots. The experimenter (CL) studied all the photographs for each horse and kept the 2 where the horse had the most and the two where the horse had the less elevated neck. In each situation (“standing”, “high walking” and “low walking” points), the values were averaged for each angle. To assess the repeatability of the measures, the angles were measured twice for each photograph. In each case, the angle values were strongly correlated (N = 93 photographs, 0.85<r_s_<0.94, p<0.00001 for each angle).

#### Terminology

Terminology in the field of vertebral/back disorders can vary from one author to another (*i.e.* [21], [36], [37]), therefore the terms in this study are defined as follows: Chiropractic evaluation is efficient in the detection of muscular stiffness and vertebral mobility, and sEMG evaluation allows the detection of musculoskeletal dysfunctions. All the disorders detected via manual palpation and sEMG evaluation will be grouped under “**back disorders**” in the rest of the following manuscript (see also [36]). The evaluations of horses’ backs were conducted outside working time to assess **chronic back disorders**. In the same way, **neck shapes** were evaluated in “every day” situations, reflecting **chronic postures** of the horse.

According to the β angle values, the horses’ neck shape will be called **concave** (if β angle positive, meaning “hollow” neck) or **round** (if β angle negative, meaning cervical flexion).

#### Data analysis

As data were not normally distributed, we used non-parametric statistical tests for the analyses. Vertebral sites were separated into 2 independent categories: sound or affected (vertebral sites could not be both “sound” and “affected”). sEMG values between these 2 categories were assessed using Mann-Whitney U-tests. Spearman correlation tests were used to assess whether chiropractic, sEMG and angle data were related to age, to detect the relations between chiropractic and sEMG evaluations, as well as the relations between sEMG measures and angle measurements of neck postures. As the different areas of the horses’ spine work together, the correlations between sEMG values at the different places along the spine were also assessed using Spearman correlation tests Finally, Chi square and Mann-Whitney U tests were used to evaluate the differences between the 2 horses’ populations. These analyses were conducted using Statistica© 10.0 software (accepted p level at 0.05).

## Results

### sEMG as a Measure for Back Disorders

The chiropractic evaluation indicated that 55% (N = 10) of our horses were severely affected, 6% (N = 1) were slightly affected and the last 39% (N = 7) were totally exempt of back disorders. The sEMG evaluation indicated that 50% (N = 9) of horses were severely affected, 11% (N = 2) were considered as slightly affected, and the last 39% (N = 7) horses were totally exempt.

**Figure 7:**
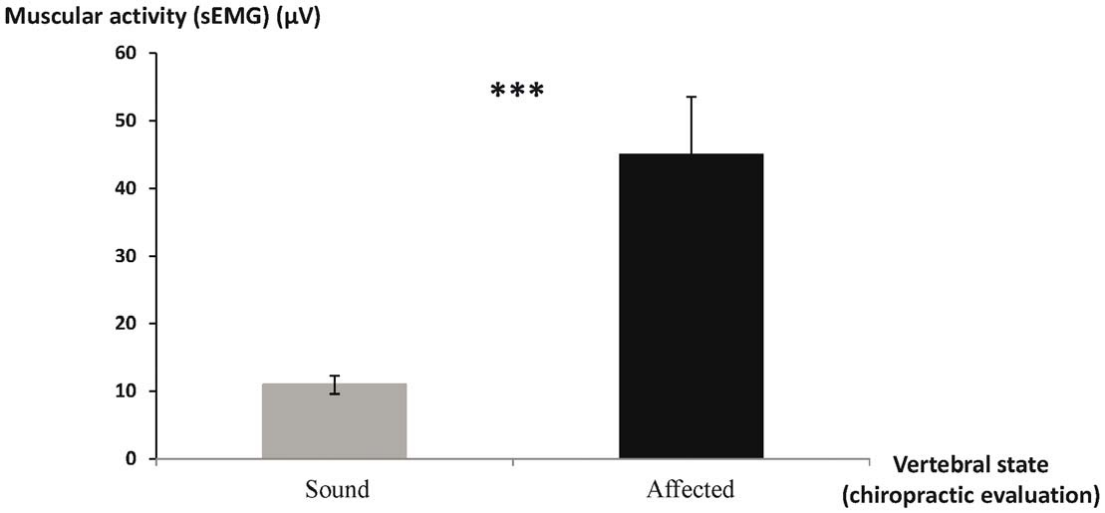
Muscular activity at the level of vertebral sites characterized as “sound” (on the left) or “affected” (on the right) by the practitioner, MW U test *** p<0.001.

The overall evaluation of the spine was highly correlated between chiropractic and sEMG evaluations, and horses with more vertebral sites affected according to manual palpation (% of affected vertebral sites) were also those with more tested sites affected according to sEMG evaluation (% of tested sites above 10 µV) (Spearman correlation tests, r_s_ = 0.82, p = 0.001) (**Fig. 7**). Moreover, both evaluations gave similar proportions of horses being severely affected (55% and 50%), slightly affected (6% and 11%) or exempt (39% and 39%) (Chi square tests, p>0.05 in all cases). In fact, the same 7 horses that were found exempt by the chiropractic evaluation were also found to be under the sEMG threshold of muscle activity, while 9 out of the 10 horses evaluated as severely affected by the chiropractor appeared so too in sEMG evaluation.

None of both evaluations found any correlation between potential back disorders and age (chiropractic: Spearman correlation test, r_s_ = −0.43, p>0.05, sEMG: Spearman correlation test, r_s_ = −0.45, p>0.05).

**Figure 8:**
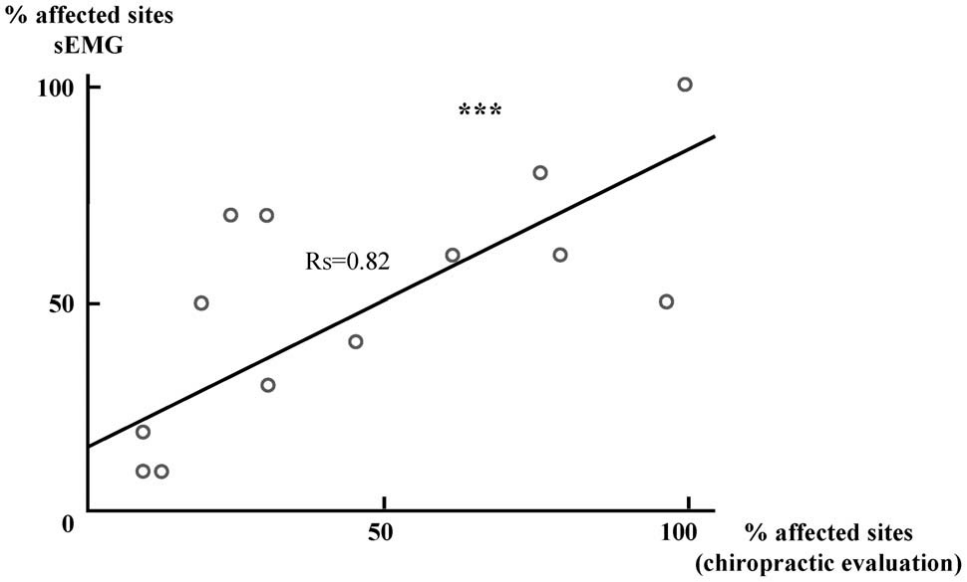
Correlations between sEMG and chiropractic evaluations. The proportion of affected tested sites per horse as evaluated by sEMG (≥10 µV) is highly correlated to the proportion evaluated for the same horse by manual palpation (N = 18horses).

Finally the sEMG values were higher at the level of vertebral sites that had been detected as affected by the chiropractic evaluations than at “healthy” sites (MW U test, N_healthy_ = 141, N_affected_ = 51, U = 1640, p = 0.001) (**Fig. 8**).

**Figure 9:**
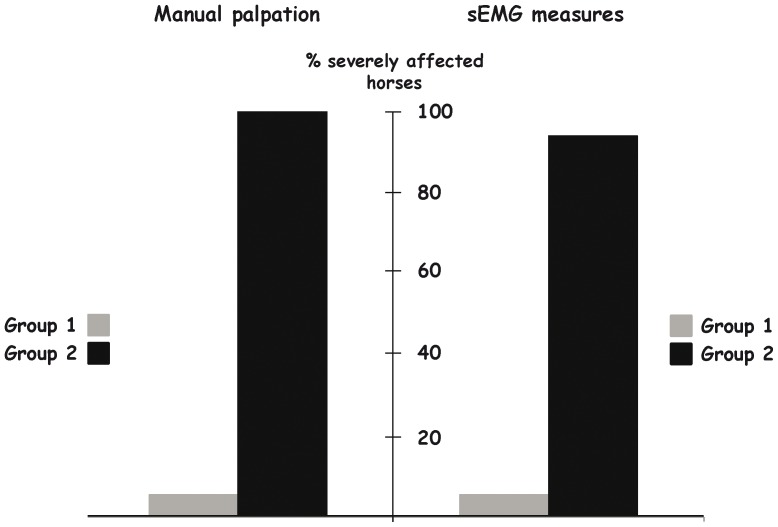
Differences between groups (1: leisure, 2: riding school) in the evaluations of back disorders. The proportion of horses evaluated as strongly affected by manual palpation (left) and sEMG evaluation (right) according to the study group (Group 1 and Group 2) is represented. Note the same important difference for both evaluations.

Both evaluations agreed in describing more severely affected horses in group 2 than in group 1 horses (Chi square tests, χ^2^ = 14.4 and χ^2^ = 10.89 for chiropractic and sEMG evaluations respectively, p<0.001in both cases) (**Fig. 9**).

### Neck Postures and sEMG Values

#### Angles and muscular measures

On the whole, horses had a more elevated neck (α angle always above the horizontal line) when standing (0.55 to 28.35 degrees,  = 12.67±1.91), whereas it varied more (above and under the horizontal line) when walking, whether the lowest (−19.9 to 2.8 degrees,  = −8.11±1.53) or highest (−16.5 to 10.55,  = −0.82±1.80) points were considered. Horses’ neck was rounder when it was at the low walking point (β angle: −12.7 to 1.75,  = −6.28±1.07), than when it was at the high walking point (−10.4 to 5.7,  = −3.5±1.1) or when standing (−10.5 to 3.35 degrees,  = −2.83±1.10). Concerning the γ angle, it was narrower when standing (angle γ: −87.65 to −66.8, = −68±7.22) and at the high walking point (−79 to −53.9,  = −67.7±1.88), than when at the low walking point (−73 to −21.8,  = −60±2.99).

Interestingly, the neck elevation during standing and walking was negatively correlated with the horses’ head-jaw angle (Spearman’s correlation tests, Standing: r_s_ = −0.5, p = 0.05; Walking: r_s_ = −0.65, p = 0.007). Thus, horses with more elevated neck postures had also more vertical heads.

sEMG values are described in **Table 1** **.** Overall, they varied little along the neck (∼13 µV, except for C3 level  = 64.75±21.71), but larger variations occurred along the spine (from 41.82±14.61 µV at T1 level to 10.48±2.33 µV at L5 level).

**Table 1:** sEMG values (µV) along the horses’ spine.

		Locations of the electrods for sEMG evaluation											
		C1	C3	C5	C7	T1	T3	T8	T10	T16	T18	L5	S1
sEMG values (µV)	range of values	1.3–45.2	1.2–300.6	1.4–42.7	1.1–56.6	0.9–203	1.4–174.6	1.9–33.5	2.1–76.3	2.9–83	1.6–42.1	0.5–40.7	0.7–30.8
Med[1–3quartile]		7.9 [3.4–23.4]	19.9 [5.3–96.2]	12.2 [3.3–17.7]	7.7 [2–16.1]	21 [4.4–60.1]	6 [2.5–31.4]	5.3 [3.9–8.4]	4.9 [3.6–23.9]	7.6 [5.1–16.9]	5.4 [2.3–7.1]	7.4 [5.7–18]	8.4 [4.4–16.9]

Range of values and Med [1^st^–3^rd^ quartiles] are represented. The values for the entire population (N = 18 horses) are presented here. Note the extreme range at C3 level.

As the horse’s spine areas cannot be considered independently, we investigated the relations between muscular activities all along the spine. The sEMG values were correlated for all cervical sites (Spearman’s correlation tests, p<0.03 in all cases) and for most back sites (Spearman’s correlation tests, T1/T3, T10, T16, L5, S1; L5/T3, T8, T10, T16, S1; p<0.05 in all cases). More interestingly still was the finding that the cervical values were correlated to those observed along the back (Spearman’s correlation test, *e.g.* C1/T3: rs = 0.57 p = 0.02; C3/T1, T3, T10, T16, L5; C5/T1, T16, L5; C7/T3, T10; p<0.05 in all cases) (**See** **Table 2** **.**).

**Table 2:** Correlations between muscular activities at different levels along the spine for the entire population.

Muscular Activity	C3	C5	C7	T1	T3	T10	T16	L5	S1
C1	rs = 0.65, p = 0.006	rs = 0,64, p = 0,007	rs = 0,66, p = 0,005		rs = 0,57, p = 0,02				
C3		rs = 0,72, p = 0,001	rs = 0,53, p = 0,03	rs = 0,66, p = 0,006	rs = 0,50, p = 0,05	rs = 0,62, p = 0,01	rs = 0,61, p = 0,01	rs = 0,63, p = 0,009	
C5			rs = 0,59, p = 0,02	rs = 0,60, p = 0,01			rs = 0,59, p = 0,02	rs = 0,68, p = 0,004	
C7					rs = 0,52, p = 0,04	rs = 0,52, p = 0,04			
T1					rs = 0,69, p = 0,003	rs = 0,57, p = 0,02	rs = 0,58, p = 0,02	rs = 0,79, p<0,001	rs = 0,59, p = 0,02
T3						rs = 0,56, p = 0,02	rs = 0,50, p = 0,05	rs = 0,65, p = 0,007	
T8								rs = 0,55, p = 0,03	
T10								rs = 0,54, p = 0,03	
T16								rs = 0,69, p = 0,003	
L5									rs = 0,60, p = 0,01

Only significant results are presented here. Note that most sites are highly correlated enhancing that the different tested sites do not “work independently” (“whole back” concept, see [68]). Spearman correlation tests are presented here.

#### Neck posture and muscular activity

A concave neck (β angle positive) was correlated with higher sEMG values (hence, muscular activity) whether the horses were standing (Spearman’s correlation test, C3: r_s_ = 0.53, C5: r_s_ = 0.57, T1: r_s_ = 0.57, T3: r_s_ = 0.75, L5: r_s_ = 0.79 and S1: r_s_ = 0.54, p<0.03 in all cases) or walking (high walking point: C1, C3, C5, C7, T3, L5, r_s_ = 0.53 to 0.59, p<0.03; low walking point: C3, C5, T1, T3, L5, S1, r_s_ = 0.57 to 0.80, p<0.02). Moreover, a wider head-jaw angle was correlated with higher sEMG values at T18 level (r_s_ = 0.53, p = 0.03) (**See** **Table 3** **.**).

**Table 3:** Correlations between neck angles measures and muscular activity (sEMG, µV) along the spine for the entire population.

	Angles	Muscular activity	C1	C3	C5	C7	T1	T3	T18	L5	S1
		α									
**STANDING**		β		rs = 0.53, p = 0.03	rs = 0.57, p = 0.02		rs = 0.57, p = 0.02	rs = 0.75, p<0.001		rs = 0.79, p<0.001	rs = 0.54, p = 0.03
		γ									
		α									
	**Highest Point**	β	rs = 0.53, p = 0.03	rs = 0.55, p = 0.03	rs = 0.59, p = 0.02	rs = 0.54, p = 0.03		rs = 0.54, p = 0.03		rs = 0.56, p = 0.03	
**WALK**		γ							rs = 0.53, p = 0.03		
		α									
	**Lowest Point**	β		rs = 0.57, p = 0.02	rs = 0.57, p = 0.02		rs = 0.75, p<0.001	rs = 0.75, p<0.001		rs = 0.80, p<0.001	rs = 0.57, p = 0.02
		γ									

α represents the neck’s elevation, β the neck’s curve and γ the M3–M5 angle. Only significant results of Spearman correlation tests are presented here. Note that the most “representative” angle is β, which is neck’s curve. The correlation reveals the the highest the angle (concave neck), the highest the muscular activity on many different tested sites.

The different measures of neck shape (angles α, β, γ) were not correlated (Sperman correlation tests, p>0.05 in all cases).

#### Comparison of 2 populations with different managements

No differences emerged between both populations concerning neck’s elevation or head-neck angle (MW U tests, p>0.05 in all cases). However, significant differences were observed between the 2 populations in terms of neck roundness, with group 1 horses presenting an angle β more negative (meaning a rounder neck) than group 2 horses, both when standing (degrees, ±es: group 1 = −7.22±1.03, group 2 = 0.08±0.73; U = 4.5, p = 0.002), and when at the high (degrees, ±es: group 1 = −7.18±1.05, group 2 =  −1.05±1.2; U = 8,5, p = 0.01) and low (degrees, ±es: group 1 = −10.5±0.89, group 2 = −3.71±0.91; U = 1, p<0.001) walking points.

**Figure 10:**
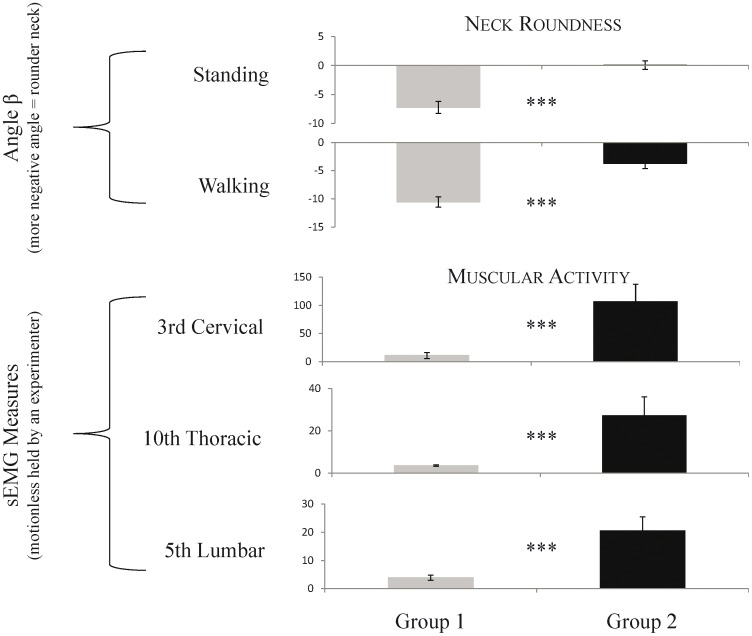
Differences in neck posture (above) and muscular activity (below) according to the study group. The more negative the angle β was, the rounder the neck was. Note that Group 1 horses have rounder necks and lower muscular activities compared to Group 2 horses. Mann-Whitney U tests, ***p<0.005.

Overall, these differences in neck postures seemed to reliably reflect differences between populations in terms of muscular activity (**Fig. 10**). Thus, group 2 horses had higher sEMG values for most tested sites (C1, C3, C5, T1, T3, T10, L5, S1; U = 0 to 10, p<0.02) (**Table 4** **.**).

**Table 4:** Representation of sEMG values differences between the 2 populations.

		Locations of the electrods for sEMG evaluation							
		C1	C3	C5	T1	T3	T10	L5	S1
sEMG values (µV, Med[1st-3rd quartile])	Group 1	2.80 [1.36–4.95]	3.84 [2.03–5.51]	1.73 [1.45–4]	3.61 [2.29–4.63]	2.86 [2.25–3.24]	3.63 [3.08–3.94]	5.02 [3.67–5.92]	5.84 [3.84–6.66]
	Group 2	19.45 [7.96–28.46]	80.34 [36.75–150.49]	10.59 [7.69–17.81]	46.33 [31.31–81.06]	24.55 [18.86–45.55]	11.44 [6.72–40.66]	14.40 [7.98–32.86]	16.52 [9.89–20.47]
MW U tests p value		U = 9, p = 0.02	U = 5, p = 0.003	U = 7, p = 0.008	U = 1, p<0.001	U = 8, p = 0.01	U = 6, p = 0.005	U = 0, p<0.001	U = 10, p = 0.02

Mediane, 1^st^ and 3^rd^ quartiles are represented as well as the results of the MW U tests. Note the large difference (3.84/80.34) in sEMG values at C3.

## Discussion

The purposes of this study were to 1) assess sEMG as a useful method for the detection of back disorders, and 2) to assess correlations between sEMG and chronic neck postures (outside working time) so as to propose neck posture as a potential visible indicator of back disorders. Elevated and concave neck postures were associated with higher sEMG values, reflecting muscular activities that correlated with back disorders, as shown by the practitioners’ evaluations. In the cases of “affected” horses, sEMG measures were higher both at the exact location of the vertebral dysfunction (assessed by the practitioner) and all along the spine. sEMG measures and neck postures therefore appeared as potentially fruitful indicators of back disorders, a major issue in this species submitted to different types of riding and management styles. Thus, comparisons of horses living in two extreme types of domestic life (including different types of work) revealed that in one (riding school), horses were more prone to have concave necks and back disorders than in the other (leisure horses).

### sEMG Measures and Back Pain

In humans, patients with back pain or lesions present higher EMG and a more important muscular fatigue than healthy people (*e.g.* [25], [40]). If EMG measure does not necessarily inform about problems’ locations, it is considered as a good indicator of their existence [25]. Cram [41] introduced the idea of “spatial dislocation of pain”, considering that EMG activation patterns are not necessarily found at the exact location of reported pain. Also, if the muscular activity right near to the lesions was not modified, LBP patients showed nevertheless increased EMG measures (Hoyt et al. 1981 in [41]), and assumed abnormal static postures [42]. Few studies were conducted on muscular activity in horses, and the ones existing mainly focused on horses’ back kinematics during movement (*e.g.* [29]). In our study, sEMG measures were increased both at the location of back dysfunctions, and also all along the spine, showing strong correlations between overall and local back dysfunctions and muscular activity. The horses’ spine has to be considered as a whole [43], and the strong correlations between muscular activities all along the spine highlights the possibility of “spatial dislocation of pain” in horses: the presence of a vertebral dysfunction in the cervical area could lead to an increase of muscular activity both in the cervical area and at the thoracic or lumbar level. Moreover, the strong relations between muscular activity at the level of C3 and all along the back seems to point out this particular site as crucial in the functioning of the horses’ back.

### Daily Postures and Muscular Activity

Some authors investigated the existing links between postural control/equilibrium and muscular activity in humans. Caneiro et al. [44] showed that sitting postures are linked with thoracic and cervical muscular activity, and an increased activity in the torso muscles was shown to disturb postural equilibrium [45]. On the other hand, postural control may be conditioned by many different factors, such as age (see [44] for a review), habitat structure (geckos: [46]), emotions (humans: [47]; anxiety: mice, [4], [48]) or physical problems (humans: [49]). Thus, aging of the sensorimotor systems involved in posture control was shown to lead to a diminution of brainstem centres controlling postures and was believed to be the main cause of deterioration in balance abilities in humans (see [50] for a review). Postures here were not related to the horses’ age, confirming earlier studies suggesting that working conditions may have a stronger impact than aging [21], [36], [37]. Habitat structure was also proved to have an effect on postural/morphological components: gecko species living in open areas exhibit more erect postures than species living in structured areas [46]. Several aspects may be involved to explain our findings: a) Horses in natural conditions graze most of the time (up to 70%, [51]), and walk with lowered head. In riding schools, they are fed in buckets fixed on the walls in elevated positions, and mostly have high doors. Thus they have to keep their head and neck high to see their environment. The postural modifications imposed by the environmental conditions may lead to chronic postural disturbances, explaining the differences between horses kept under semi-natural conditions and riding school horses. b) Global living conditions may impact on horses’ stress level (social isolation: [19], [52], feeding/foraging activity: [20], [53]) which could lead to muscular stiffness or tensions. Anxiety in mice leads to flatter postures, whereas calmness leads to rounder postures [4] and distressed adolescents showed more uneven shoulder height than non distressed ones [54]. Our study confirms earlier findings that group living and grazing opportunities led to horses with more “neck roundness” [11]. c) Riding techniques are also certainly important. In humans, the suppression of emotions required in some kinds of jobs may lead to health and especially musculoskeletal disorders [55], [56], [57]. Overall, imposed working postures may lead to various muscular (*e.g.* Children at school: [58]; computer workers: [59]; employee of fiscal office: [60]) or musculoskeletal (Dentists: [61]; see [62] for a review) dysfunctions. Thus, postures can be considered in humans as an indirect measure of back disorders. In horses, the use of inappropriate punishment and of contradictory orders for example may lead to increased emotionality, or even to pathological behaviours [17], [18], [63]. A study conducted on a large sample of animals showed that sport horses are more emotive than leisure ones, suggesting that stronger working constraints actually impact on horses’ behavioural reactions [64]. Physical reasons may explain such over reactions: Cook [65] suggested that pain/discomfort linked to the inappropriate use of bits could lead to resistances and fight behaviours inducing neck rigidity and gait disturbances. Inappropriate hands actions were also suggested to induce fear/escape reactions in horses, such as raising head and neck [65], [66]. The repetition of inappropriate hands and or reins actions could lead to chronic postures. Thus, a recent study highlighted a strong link between riding techniques, postures at work and chronic vertebral disorders [12]. In riding schools where beginners have high hands and short reins, horses tend to have higher (and more concave) neck postures at work while also exhibiting more chronic vertebral disorders [12]. In this study, group 1 horses were used mostly for leisure activity, ridden with low hands, long and slacken reins which differs from most riding lessons practices [12]. In our study, measures were taken on extensor muscles of the neck and the back of the horses, muscles that are linked together and are responsible of skeleton integrity [67]. Indeed, a lowering of the neck leads to an increase of the gap between thoracic spinal processes, and consequently to an extension of the *longissimus dorsi* and of the entire spine, and to a global “round” posture of the horse [43]. Thus, muscular dysfunction (modification of the basal tonus) could reveal or predict more severe lesions.

Several factors, such as age, body fat, skin resistance or fear can modulate sEMG results. In this study, horses all presented the same corporal state (optimal), measure were conducted outside any disturbances and no fear reactions were observed (see also Fig. 2 & 5). Moreover, neither age, nor breed had any effect on the muscular activity recorded, suggesting that if any of these parameters had any effect, it should have been minimal. Surface EMG measures are recognized as indirect measures of back pain, indicating the existence of vertebral/musculoskelettal disorders more than their localizations (see [25]).

This study led to the identification of key postural elements, allowing indirectly the detection of potential back disorders. The importance of the muscular activity at the level of C3 and of the β angle in neck shape points out neck “roundness” (position of M2 compared with the line between the neck basis and the head/neck joint) as a reliable indicator of back disorders, easy to evaluate in field conditions. This is of a considerable interest in a fundamental point of view, highlighting the accuracy of using postural elements to evaluate the animals’ general state and has important implications as a tool for animals’ welfare evaluation.
